# PRECEDE-PROCEED model for discharge preparation in first-cycle colorectal cancer chemotherapy: a quasi-experimental study

**DOI:** 10.3389/fonc.2026.1734836

**Published:** 2026-03-31

**Authors:** Xiuhua Chen, Huangjing Chen, Wangjuan She, Yeping Ge, Bihe Qiu, Wei Zhu, Haiyan Gu

**Affiliations:** 1 Department of Oncology, Affiliated Hospital No. 2 of Nantong University, Nantong First People’s Hospital, Nantong, China; 2 Department of Ultrasound, Affiliated Hospital of Nantong University, Nantong, China; 3 Department of Nursing, Affiliated Hospital No. 2 of Nantong University, Nantong First People’s Hospital, Nantong, China

**Keywords:** Colorectal cancer, discharge preparation, first cycle of chemotherapy, health education, PRECEDE-PROCEED model

## Abstract

**Objective:**

The aim of this study was to evaluate the impact of discharge preparation guided by the PRECEDE-PROCEED model of health education in patients with colorectal cancer undergoing their first cycle of chemotherapy.

**Methods:**

A total of 168 patients with colorectal cancer receiving their first chemotherapy cycle in the Department of Medical Oncology between January and August 2025 were assigned to either the control group (*n* = 82, routine health education) or the experimental group (*n* = 86, PRECEDE-PROCEED intervention) based on admission time. Outcomes including readiness for hospital discharge, quality of discharge guidance, home care experience, and readmission rate were compared between the groups.

**Results:**

Patients in the experimental group demonstrated significantly higher mean scores for total hospital discharge readiness (100.70 ± 3.80 vs 85.77 ± 3.53, *p* < 0.001), total discharge guidance quality (141.12 ± 3.59 vs 134.28 ± 4.21, *p* < 0.001), and home care experience on the 5th and 10th days after discharge (28.63 ± 1.95 vs 24.73 ± 2.63, *p* < 0.001 and 31.19 ± 2.12 vs 26.49 ± 1.86, *p* < 0.001) compared with the control group. No statistically significant difference was observed between the groups in terms of unplanned readmission within 3 weeks (*p* > 0.05).

**Conclusion:**

Health education based on the PRECEDE-PROCEED model enhanced discharge readiness, the quality of discharge guidance, and the home care experience of patients with colorectal cancer receiving their first cycle of chemotherapy, although it did not significantly reduce unplanned readmission within 3 weeks. These findings suggest that integrating the PRECEDE-PROCEED model into discharge preparation may serve as a low-cost, high-impact intervention to improve transition safety and patient experience in first-cycle CRC chemotherapy.

## 1 Introduction

Colorectal cancer (CRC) represents a major global health challenge. According to 2024 American Cancer Society statistics, CRC ranks as the third most common malignancy in both sexes, accounting for 7.6% of new cancer cases worldwide (1). In China, the 2022 National Cancer Center report documented continued increases in both incidence and mortality (2, 3), posing substantial threats to public health.

Health authorities now aim to shorten hospital stays and save resources. As a result, patients are discharged shortly after treatment completion. Chemotherapy remains the main treatment for colorectal cancer, but its side-effects can impair prognosis and quality of life (4, 5). If patients leave hospital without adequate preparation, they may struggle to manage these side-effects at home. Many feel uncertain and unsafe (6). First-time chemotherapy patients are especially vulnerable. Limited knowledge triggers anxiety, fear and even refusal of further treatment. Concerns regarding complications, including fatigue, vomiting, and diarrhea, may further exacerbate fear and result in treatment refusal (7). Systematic discharge preparation is therefore essential to ensure smooth transitions from hospital to home, enhance patients’ ability to manage treatment side effects, and improve post-discharge quality of life.

Prior studies have indicated that comprehensive discharge preparation improve the ability of patients to manage health challenges encountered after discharge (8–10). Fenwick introduced the term in 1979. It denotes the systematic, individualized support given by health-care professionals during admission. The goal is to ensure that patients and caregivers can move safely from hospital to home and continue effective care (11). Health education is a key component. It strengthens self-management and is highly demanded by oncology patients and families (12, 13).

The PRECEDE-PROCEED model of health education was developed by American health educator Lawrence Green. This model adopts a multidisciplinary perspective and emphasizes a “results-oriented” backward design approach. By analyzing predisposing, enabling, and reinforcing factors that influence health outcomes, it provides a systematic framework for developing health interventions. Today, it is one of the most widely used and authoritative models in the field of health education (14, 15). Compared with the Transitional Care Model and Social Cognitive Theory, the PRECEDE-PROCEED model offers distinct advantages. While the Transitional Care Model emphasizes integrated care, it lacks a systematic diagnostic tool to assess patient needs (16). Social Cognitive Theory is effective in explaining individual behavior change but is limited in guiding the design and evaluation of comprehensive, population-level interventions (17). In contrast, the PRECEDE-PROCEED model combines in-depth needs assessment with broad implementation and evaluation capabilities, making it particularly suitable for designing a comprehensive nursing intervention in this study. Based on this model, a clear *a priori* logical framework was established. In the PRECEDE phase (diagnostic phase), predisposing, enabling, and reinforcing factors influencing patients’ outcomes during initial chemotherapy were analyzed to inform the design of peri-chemotherapy interventions. In the PROCEED phase (implementation phase), targeted nursing interventions were delivered in stages, aiming to improve these key factors. Ultimately, the goal was to enhance patients’ readiness for discharge, the quality of discharge guidance, and their home-care experience. This logic model ensures both theoretical rigor and the specificity of the intervention measures. Although used in discharge preparation, no study has applied it to first-cycle colorectal-cancer chemotherapy patients (18–20). We therefore conducted this study to test PRECEDE-PROCEED-based education in this population.

## 2 Participants and methods

### 2.1 Study participants

A quasi-experimental design was used, and convenience sampling was utilized to recruit patients with colorectal cancer who underwent first chemotherapy in the medical oncology ward of a Grade-A tertiary hospital in Jiangsu Province between January 2025 and August 2025. The primary outcome measures included readiness for hospital discharge, discharge instruction quality, home health care experience, and unplanned readmission rate. Sample size calculation was based on the primary outcome, patient readiness for hospital discharge, measured using the Chinese version of the Readiness for Hospital Discharge Scale. The anticipated effect size was derived from data reported in a similar discharge education intervention study (21). We estimated that implementation of the PRECEDE-PROCEED–based intervention would increase the mean Readiness for Hospital Discharge Scale score in the intervention group by 5.6 points compared with the control group, with an assumed standard deviation of 4.6 points. A two-sided significance level of α = 0.05 and a statistical power of 1 − β = 0.80 were specified for the sample size calculation. The results indicated that *N1* = *N2* = 38. After accounting for a 10% attrition rate, a minimum of 42 patients per group were required. A total of 168 patients were ultimately included. The sample size calculation was based on the primary outcome (RHDS score) without adjustment for multiple comparisons. Given the exploratory nature of secondary outcomes (discharge teaching quality, home care experience, and readmission rate), we prioritized statistical power for the primary endpoint. Secondary outcomes are reported with unadjusted p-values, acknowledging the increased risk of Type I error. We considered this approach appropriate given the study’s focus on evaluating a comprehensive intervention where effects on multiple related outcomes are biologically plausible and clinically informative.

Patients who received their first cycle of chemotherapy between January and April 2025 (*n* = 82) were assigned to the control group, while those who received it between May and August 2025 (*n* = 86) were assigned to the experimental group.

The inclusion criteria were: (1) age between 18 and 70 years; (2) receiving the first cycle of an oxaliplatin-based chemotherapy regimen within 28 days after radical resection for colorectal cancer; (3) pathological clinical stage II–IV; (4) Eastern Cooperative Oncology Group Performance Status (ECOG-PS) score 0–2; (5) able to use the intelligent nursing platform (a smartphone application, hereinafter referred to as “the platform”) after training (this criterion may introduce selection bias by excluding patients without smartphone access or digital literacy, potentially limiting applicability to older adults, lower socioeconomic groups, or rural populations. During the recruitment period, approximately 8% of eligible patients were excluded due to inability to use the platform); and (6) willing to participate and having signed informed consent.

The exclusion criteria were: (1) inability to understand the medical condition; (2) presence of significant visual, language, or comprehension disorders, or mental illness; (3) diagnosis of severe cardiovascular or cerebrovascular disease; (4) concomitant other malignant tumors or recurrent/metastatic disease; and (5) participation in other interventional studies.

This study was reviewed and approved by the Ethics Committee of the hospital (Approval No. B2025-KT259-01).

Randomized controlled trial was not feasible in this clinical setting due to ethical concerns regarding withholding an evidence-based intervention from control group patients once the PRECEDE-PROCEED program was implemented. Additionally, the phased rollout (January-April 2025 for control, May-August 2025 for experimental) was necessitated by the need for structured staff training and system preparation (the platform configuration, educational material development) before intervention implementation. We monitored potential temporal confounders throughout the study period, including: (1) chemotherapy protocols and dosing regimens (no changes); (2) nursing staff composition and experience levels (stable personnel); (3) hospital discharge policies (unchanged); and (4) seasonal disease patterns (no influenza outbreaks or unusual infectious disease events during either period). These factors remained constant, minimizing the risk of temporal bias.

### 2.2 Study methods

#### 2.2.1 Intervention methods for the control group

On admission, the responsible nurse provided routine education on disease, chemotherapy, vascular access, and stoma care. On the day of discharge, patients received additional instructions on adverse-reaction self-management, medication, follow-up, port maintenance, and stoma care. Two hours prior to discharge, patient understanding of the educational content was verified by a cancer specialist nurse using the feedback method. In addition, readiness for hospital discharge and the quality of discharge instructions were assessed. Follow-up telephone interviews, each lasting 5 to 8 minutes were conducted on the 5th and 10th days after discharge. These interviews addressed chief complaints, blood test re-examinations, medication guidance, port maintenance, stoma care, dietary and rest considerations, and other home-based self-care practices. The occurrence of chemotherapy-related adverse reactions was recorded, and questions by patients were answered.

#### 2.2.2 Intervention methods for the experimental group

Discharge preparation based on the PRECEDE-PROCEED model was provided to the experimental group. Predisposing, enabling, and reinforcing factors associated with the first cycle of chemotherapy were assessed by oncology staff. From prior evaluations of hospital discharge readiness and discharge preparation quality, common challenges among patients undergoing their first cycle of chemotherapy were summarized. Cancer specialist nurses then implemented phased interventions through health education and nursing using the PRECEDE-PROCEED model.

**Figure 1:**
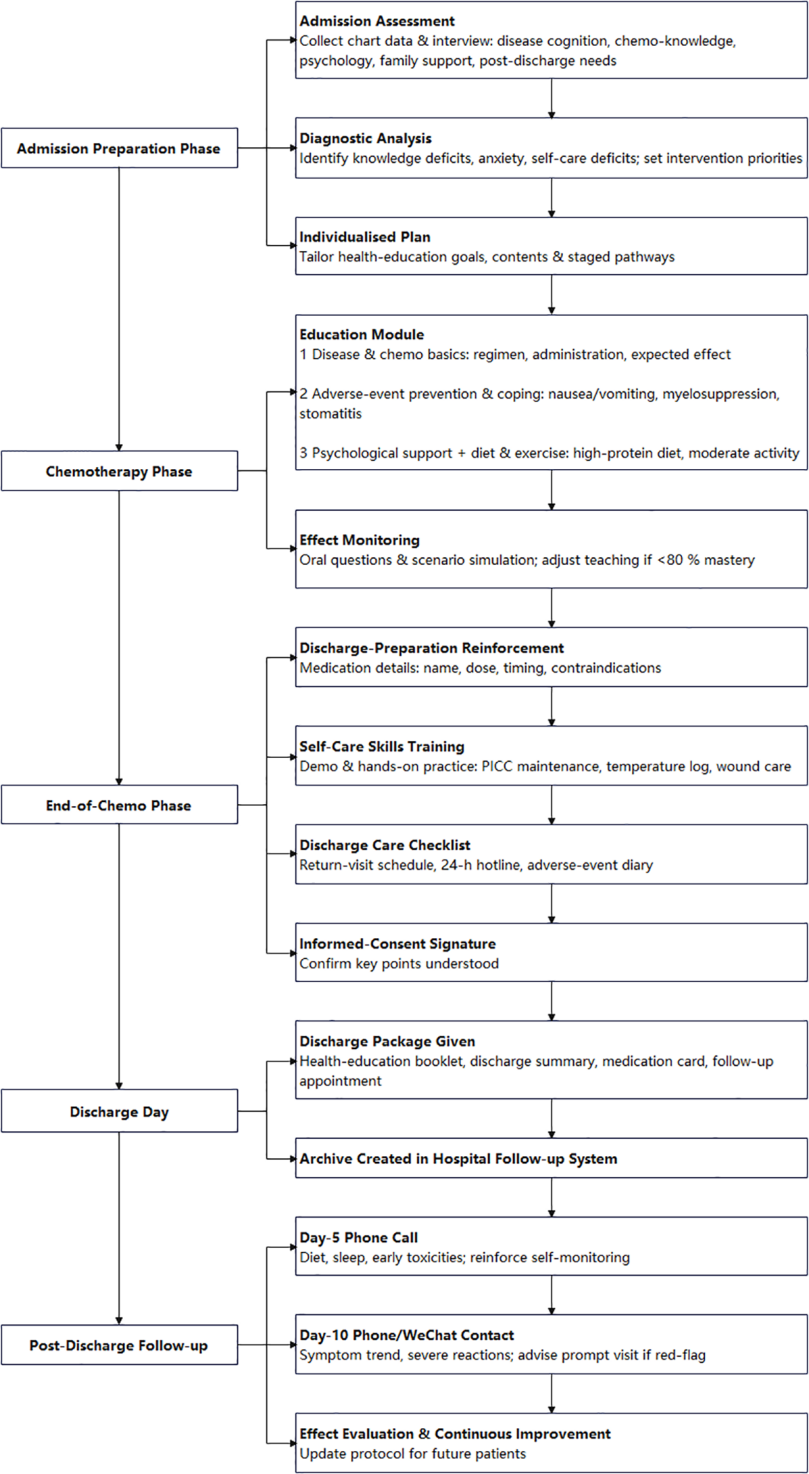
PRECEDE-PROCEED model-based discharge preparation intervention framework for patients receiving first-cycle colorectal cancer chemotherapy.

Health education based on the PRECEDE-PROCEED model was implemented in two phases (**Figure 1**). PRECEDE phase (admission): Comprehensive assessment of disease cognition, chemotherapy knowledge, psychological status, and family support, followed by individualized goal-setting. PROCEED phase: Stage-based interventions during chemotherapy (knowledge, adverse reaction management, psychological support), completion (medication guidance, skills training with demonstration-practice-feedback, discharge checklist), and post-discharge follow-up (telephone/WeChat on days 5 and 10). Process and outcome evaluations were conducted continuously.

##### 2.2.2.1 Establish a discharge preparation team

The 11-member team comprised a department director, head nurse, three oncology specialist nurses, two master’s students, a dietitian, a psychotherapist, an IV-therapy nurse, a stoma therapist, and an information engineer. The director oversaw treatment; the head nurse coordinated the project; specialist nurses led assessment and education; the information engineer maintained the data platform.

##### 2.2.2.2 Personnel training

###### 2.2.2.2.1 Nurse training and certification

To ensure intervention fidelity, the head nurse conducted standardized training for all research nurses. Prior to participant enrollment, each nurse completed a 5-hour structured curriculum and achieved ≥90% on a competency assessment to receive certification.

The training curriculum included: (1) Application of the PRECEDE-PROCEED model in health education; (2) Current status and recent advances in discharge preparation services; (3) Key nursing considerations and safety management during the first cycle of chemotherapy for colorectal cancer; (4) Essential knowledge on stoma care and implantable venous access port maintenance; (5) Practical training, including administration of the Readiness for Hospital Discharge Scale, use of the chemotherapy complication intelligent module, and standardized operational procedures. All teaching sessions were guided by validated checklists developed based on the PRECEDE-PROCEED framework. To ensure intervention fidelity, 20% of education sessions were randomly audio-recorded and reviewed by the principal investigator. A standardized fidelity checklist was used to assess two core domains: (a) adherence to protocol (delivery of all prescribed educational components at specified time points), and (b) quality of communication (clarity of explanation, patient engagement, and appropriate response to patient questions). Sessions were rated on a 3-point scale (1 = needs improvement, 2 = satisfactory, 3 = excellent), with scores ≥2 considered acceptable fidelity.

###### 2.2.2.2.2 Patient education

The patient education component consisted of structured, stage-specific interventions: (1) Individual education sessions: Three structured one-on-one teaching sessions were conducted at key time points: upon admission, during the first chemotherapy cycle, and prior to discharge. Each session lasted 20–30 minutes. (2) Digital platform support: Through the platform, patients received one daily educational message related to chemotherapy care. On post-discharge days 5 and 10, the platform prompted patients to complete two assessments: chemotherapy-related adverse symptom reporting and home care experience evaluation. (3) Intervention duration: The intervention covered one complete chemotherapy cycle (21 days).

##### 2.2.2.3 Clinical implementation of the intervention program

Health education based on the PRECEDE-PROCEED model was implemented by oncology nurses in two phases: the Behavioral Diagnosis PRECEDE phase and the Health Intervention PROCEED phase.

1. The PRECEDE phase involved assessments at five levels: social, epidemiological, behavioral environment, educational organization, and management policies. Specialist nurses built patient files on the platform and performed face-to-face interviews to assess sociodemographic data, disease understanding, acceptance of chemotherapy, adverse-reaction knowledge, and family support.

The educational organization evaluation, considered the core of the PRECEDE-PROCEED model, involved comparing prior hospital discharge readiness and discharge guidance quality, followed by summarizing the predisposing, enabling, and reinforcing factors influencing patients undergoing their first cycle of chemotherapy.

Predisposing factors included a lack of correct perception of chemotherapy among most patients and their families, concerns about adverse effects, unclear knowledge of self-care after discharge, overdependence on family members, low self-efficacy, and limited acceptance of health education content among patients with lower knowledge levels.

Enabling factors included complex and unfocused health education content, limited and monotonous educational materials, delivery methods that were difficult for patients to accept, insufficient communication with patients and families, and unclear criteria for evaluating the education process and quality.

Reinforcing factors included limited patient motivation to actively learn, insufficient supervision by medical staff, inadequate family support and supervision, and the absence of targeted follow-up guidance during chemotherapy intervals.

For management policy evaluation, health education plans and interventions were developed by oncology nurses, with continuous monitoring and management conducted through the platform after discharge.

2. The PROCEED phase comprised of four parts: targeted intervention implementation, process evaluation, impact evaluation, and outcome evaluation (22). During the first hospitalization for chemotherapy, one-on-one visits were conducted, and patients were assessed based on diagnosis results and treatment stages. A health education plan and implementation approach were jointly developed by the research team, using the health education plan list as a reference and finalized through team discussions, as presented in **Table 1**. Patients were followed up by telephone on two occasions after discharge, and real-time communication was maintained via the platform.

**Table 1:** Health education plan and implementation based on the PRECEDE-PROCEED model for patients receiving their first cycle of chemotherapy in the experimental group.

Intervention stage	Categorical factors	Health education intervention PROCEED implementation phase
Admission preparation stage	Predisposing factors	1. Description of the hospital environment, introduction of the research team, and detailed explanation of the health education program content. 2. Provision of colorectal cancer-related knowledge, including etiology, clinical manifestations, treatment principles, relevant examinations, and key testing points. 3. Patient assessment upon admission and establishment of the discharge preparation service record within the intelligent nursing information platform.
	Enabling factors	1. Information on the chemotherapy cycle and the expected length of hospital stay, and provide psychological preparation for discharge. 2. Instruct the patient on using the Shiguang Zhijian mobile application, including setting alarm reminders to encourage completion of “My Visit Diary” for symptom recording and self-management.
	Reinforcing factors	1. Encourage patients to adopt a positive attitude toward their disease and enhance confidence in treatment outcomes. 2. Facilitate participation in a WeChat patient support group to promote peer support and mutual assistance.
		3. Provision of psychological counselling services for patients who require additional support.
Chemotherapy stage	Predisposing factors	1. Provide video-based education for patients and caregivers covering precautions related to safety, diet, exercise, and rest. 2. Explain the condition of the patient, chemotherapy regimen, expected effects, possible adverse reactions, and options for intravenous access. 3. Distribute the “Colorectal Cancer Peri-chemotherapy Health Education Manual for Patients and utilize the Precautions Card for Chemotherapy Pump or Small Prescription for Oral Chemotherapeutic Drugs” to enhance understanding of colorectal cancer and its treatment. 4. Provide individualized guidance on stoma care and indwelling venous catheter management according to patient-specific needs.
	Enabling factors	1. Review the platform operation video, including instructions on the chemotherapy knowledge base sub-module, adverse reaction self-assessment, and platform interaction, until the patient can operate independently. 2. Instruct patients on self-monitoring of vital signs and observation for the occurrence of acute adverse reactions.
	Reinforcing factors	1. Encourage patients and caregivers to participate in colorectal cancer knowledge lectures to enhance understanding of the disease. 2. Involve caregivers in supervising patients to modify unhealthy habits and establish healthy lifestyle practices.
Chemotherapy completion stage	Predisposing factors	1. Provide educational information on granulocyte colony-stimulating factor, including explanations of the differences between long-acting and short-acting injections and potential adverse reactions.
	Enabling factors	1. Guide patients in self-assessment of gastrointestinal reactions via the platform and provide tailored prescriptions to reduce such reactions, including acupressure prescriptions, methods for maintaining oral moisture, and constipation prevention; situationally demonstrate common self-management errors for correction by patients and caregivers. 2. Instruct the patients to undergo timely reexamination of blood routine and liver function, and provide information regarding the frequency of blood tests, grading of bone marrow suppression, and self-protection strategies after chemotherapy. 3. Provide access to the stoma care platform of the hospital to facilitate understanding of stoma management and available assistance. 4. Collaborate with patients to develop individualized nutritional plans and exercise programs, utilizing tools such as WeChat pedometers and exercise watches to support completion of daily exercise goals.
	Reinforcing factors	1. Guide patients to positively address changes in body image by providing psychological counseling and support. 2. Encourage caregivers to offer comfort and accompany patients throughout the treatment process.
Discharge stage	Predisposing factors	1. Evaluate the effectiveness of PROCEED care measures at the end of treatment and provide additional guidance if necessary to ensure completion of discharge preparation services. 2. Distribute a questionnaire before discharge, conducted by the oncology specialist nurse, to assess the patient’s readiness for hospital discharge.
	Enabling factors	1. Apply the explanation–demonstration–feedback method based on the guided drug checklist, covering oral cytotoxic drugs, targeted antitumor drugs, and other medications; instruct patients in correct administration methods, precautions, and ensure participation of at least one caregiver. 2. Conduct simulations for filling in laboratory test results, inquiry of issues, and interaction with medical staff via the platform. 3. Simulate home-based activities to help reduce fatigue. 4. Provide information regarding possible adverse reactions, indications for seeking medical attention, and relevant contact numbers. 5. Ensure the responsible nurse confirms the completion of all discharge preparation services for the patient.
	Reinforcing factors	1. Guide patients in the transition from hospital to home while providing positive psychological support.
The 5th and 10th days after discharge	Predisposing factors	1. Cancer specialist nurses assess patients’ rest, activity, and diet during the interval between chemotherapy, evaluate the degree of chemotherapy toxicity, review reexamination results, and check for abnormalities at the care of the implanted venous access port, providing targeted health guidance accordingly; with the support of an the platform, send reminders and conduct two telephone follow-ups on the 5th and 10th days after discharge.
	Enabling factors	1. Oncology specialist nurses send reminders twice weekly and adjust the health education program promptly according to patients’ conditions. 2. Patients use the platform to access self-care knowledge, record adverse reactions, and schedule offline or home visits. 3. Patients access relevant information from the platform knowledge base at any time and receive peer education support through the interactive online consultation module.
	Reinforcing factors	1. Patients logged into the public interface for home care self-assessment on the 5th and 10th days after chemotherapy, and the system automatically generated scores, which were sent to the home page of the fixed computer intelligent system and the mobile the platform of the postgraduate nurses. 2. Oncology nurses explored difficulties encountered by patients during home care and provided targeted interventions directly to the patient’s mobile terminal. 3. Patients accessed relevant knowledge from the platform knowledge base at any time or received peer education support through the interactive online consultation module. 4. Compliant patients were invited to share their experiences and achievements.

#### 2.2.3 Quality-assurance procedures

The two outcome assessors were oncology specialist nurses involved in the intervention delivery; therefore, they were not blinded to group allocation. This may introduce detection bias, as assessors might unconsciously favor the experimental group when administering scales or recording responses. To minimize this bias, we implemented: (1) standardized, scripted assessment protocols with specific wording for all scale items; (2) independent double-data entry with discrepancy resolution; and (3) random audio-recording of 20% of assessments for fidelity monitoring. Nevertheless, the lack of independent blinded assessors remains a methodological limitation.

To guarantee data quality throughout the trial, the following measures were instituted:

1. Two certified oncology specialist nurses, who had received uniform training, acted as outcome assessors for the entire study.
2. Before discharge, the two nurses explained the Readiness for Hospital Discharge Scale and the Quality of Discharge Teaching Scale to patients and their caregivers in a standardized manner to avoid heterogeneous interpretations.
3. Any uncertainties raised by participants during the interview were clarified immediately; doubtful answers were verified on the spot.
4. After discharge, the intelligent nursing platform automatically sent text reminders at 20:00 every day, prompting patients to complete the home-experience questionnaire. If submission was delayed > 24 h or the data were obviously deviant (e.g., all items scored identically), one of the two nurses telephoned the patient to check and correct the record.

### 2.3 Evaluation indicators

1. General data: Data on sex, age, marital status, education level, family care, disease diagnosis, disease stage, chemotherapy regimen, Eastern Cooperative Oncology Group (ECOG) performance status score, and the presence of chronic comorbidities, along with other relevant characteristics were collected.

2. Readiness for Hospital Discharge Scale: The original version of Readiness for Hospital Discharge Scale was developed by Weiss and Piacentine (23). The 12-item Chinese version of the scale (Cronbach’s α = 0.89) covers personal status, adaptability, and expected help; higher scores indicate greater readiness. The scale showed dimension-specific coefficients ranged from 0.80 to 1.00, and the content validity index (CVI) was 0.88 (18).

3. Quality of discharge teaching scale: The quality of discharge teaching scale, translated and revised by Wang et al., comprised of 3 dimensions: required content (a1–a6 items), obtained content (b1–b6 items), and the effectiveness of discharge guidance skills (12 items) (24). The required content and obtained content dimensions were paired into 6 groups for comparison, with the difference in scores reflecting whether discharge instruction quality met the care needs of patients. The total score was calculated from the obtained content and effectiveness of guidance skills dimensions. Items were scored on a 0 to 10 scale, with higher scores indicating higher quality of discharge guidance. The reliability of the scale was high, with a Cronbach’s α coefficient of 0.924 for the overall scale, 0.882 to 0.935 across dimensions, and a CVI of 0.98.

4. Patient Assessment of Care for Transitions-Medicare (PACT-M), developed by Oikonomou et al., is designed to evaluate the quality and safety of care during the transition from hospital to home and includes two parts (25): PACT-M1 (9 items), which evaluates patients’ experiences with transition preparation, and PACT-M2 (8 items), which assesses home health care following discharge, for a total of 17 items.

In this study, PACT-M2 was used to evaluate the home health care experience of patients through telephone follow-up on the 5th and 10th days after discharge. Items were rated on a 5-point Likert scale ranging from “Strongly Disagree” to “Strongly Agree,” with an option of “Not Applicable,” which was excluded from the total score. Higher total scores indicated greater safety of self-care at home. The scale demonstrated strong reliability, with a Cronbach’s α coefficient of 0.92. Liu et al. translated the scale into Chinese, and the Cronbach’s α coefficient for PACT-M2 was 0.793 (26).

### 2.4 Data collection

Oncology nurses assessed the readiness of patients for hospital discharge and the quality of discharge instructions within eight hours of admission. Two hours prior to discharge, both readiness for hospital discharge and the quality of discharge instructions were reassessed, and relevant data were recorded and summarized. During the post-discharge period, patients in both groups reported adverse reactions to chemotherapy and home health care experiences on the 5th and 10th days through an the platform. Each day at 20:00, two oncology nurses verified the completeness and accuracy of the submitted data; significant deviations were addressed promptly through direct patient contact. Postgraduate nursing students compiled and summarized the data on a weekly basis, including patient demographic information, records of adverse reactions to chemotherapy, and home health care experiences.

### 2.5 Statistical methods

Data were analyzed using SPSS version 26.0 (IBM Corp., Armonk, NY, USA). Normal data are presented as mean ± SD ( $\bar{x}$*± s*) and compared with independent-samples t tests; categorical data are compared with χ² tests. Significance was set at p < 0.05. For the PACT-M2 scale, the frequency and handling of “Not Applicable” responses were documented. Items marked as ‘Not Applicable’ were handled as follows: (1) if >20% of items in any dimension were ‘Not Applicable’, the questionnaire was excluded from validity analysis due to incomplete data; (2) if ≤20% of items were ‘Not Applicable’, missing values were imputed using the individual’s mean score for that dimension, with explicit notation in the dataset.

## 3 Results

### 3.1 Comparison of general data between two groups

For the PACT-M2 scale, participants who had 1–2 items marked as “Not Applicable” were subjected to mean imputation within the respective dimension. No participant exceeded the 20% threshold for questionnaire exclusion. The primary results was compared with a complete-case analysis (excluding participants with any “Not Applicable” responses). Findings remained consistent, supporting the robustness of the imputation approach.

During the study, 3 patients in the experimental group and 2 patients in the control group were lost to follow-up. Among these cases, 2 patients were transferred to other hospitals for treatment, and 3 patients withdrew for other reasons. A total of 168 valid cases were included, comprising of 86 patients in the experimental group and 82 patients in the control group. In both groups, a higher proportion of patients were male, had completed junior high school education, and were married. The mean age of patients in the experimental group was 59.07 ± 5.36 years, and in the control group, it was 59.44 ± 9.43 years.

Most patients diagnosed with colorectal cancer had a disease stage of pT2N1b, and received an oxaliplatin plus fluorouracil chemotherapy regimen. The mean length of hospital stay was 3.21 ± 0.46 days in the experimental group and 3.20 ± 0.43 days in the control group. Many patients used health insurance to cover healthcare costs. The mean ECOG performance status score was 1.41 ± 0.62 in the experimental group and 1.43 ± 0.63 in the control group, and most patients had no chronic comorbidities. All patients had implanted venous access ports and reported good family support. There were no statistically significant differences in general characteristics between the two groups (*p* > 0.05), as presented in **Table 2**.

**Table 2:** Comparison of baseline characteristics between experimental and control groups.

Category		Experimental group	Control group	Test statistic	*p* value
		(*n* = 44)	(*n* = 42)		
Sex	Male	50	53	0.746	0.388
	Female	36	29
Age		59.07 ± 5.36	59.44 ± 9.43	0.31	0.757
Education level	Illiteracy	2	2	9.855	0.079
	Primary school	24	16		
	Junior high school	56	51		
	High school	0	6		
	College	4	5		
	Bachelor’s degree or above	0	2		
	Married	78	69	5.038	0.081
Marital status	Divorced	6	13		
	Widowhood	2	0		
Diagnosis	Colorectal cancer	42	41	0.034	0.983
	Rectal cancer	35	33		
	Duodenal cancer	9	8		
Disease stage	pT2N1a	4	4		
	pT2N1b	28	27		
	pT3N1	24	21	0.123	0.998
	pT3N2	4	4		
	pT4bN2a	26	26		
Chemotherapy regimen	Oxaliplatin	4	6	1.226	0.747
	Oxaliplatin+Irinoteca	10	14		
	Oxaliplatin+Fluorouracil	60	60		
	Oxaliplatin+Capecitabine	12	2		
Length of hospital stay		3.21 ± 0.46	3.20 ± 0.43	-0.206	0.837
Payment method of medical expenses	Health insurance	76	68	1.016	0.313
	Self-pay	10	14		
ECOG score		1.41 ± 0.62	1.43 ± 0.63	0.206	0.837
Whether combined chronic disease	Yes	9	8	0.129	0.719
	No	73	78		

pT2N1a: tumor invades the muscularis propria with one regional lymph node metastasis; pT2N1b: tumor invades the muscularis propria with two to three regional lymph node metastases; pT3N1: tumor penetrates the muscularis propria to reach the subserosa, or invades adjacent colorectal tissues without peritoneal coverage, with one to three regional lymph node metastases; pT3N2: tumor penetrates the muscularis propria to reach the subserosa, or invades adjacent colorectal tissues without peritoneal coverage, with ≥4 regional lymph node metastases; pT4bN2a: tumor directly invades or adheres to other organs/structures, with four to six regional lymph node metastases.

### 3.2 Comparison of readiness for hospital discharge and discharge instruction quality between groups

Prior to the intervention, no statistically significant differences were observed in the readiness for hospital discharge or discharge instruction quality dimension scores between the two groups (*p* > 0.05). Following the intervention, the experimental group demonstrated significantly higher scores in all dimensions of readiness for hospital discharge and discharge instruction quality compared with the control group (*p* < 0.05), indicating improved preparedness and discharge guidance. Post-intervention, the effect size for readiness for hospital discharge was large (Cohen’s d = 4.067, 95% CI [3.533, 4.597]). Similarly, the effect size for discharge teaching quality was large (Cohen’s d = 1.749, 95% CI [1.391, 2.103]). Detailed results are presented in **Table 3**.

**Table 3:** Comparison of readiness for hospital discharge and quality of discharge instructions between experimental and control groups.

Category	Number of cases	Readiness for hospital discharge				Quality of discharge instructions		
		Personal status	Adaptability	Expectedness support	Total score	Content actually obtained	Guiding skills and effects	Total score
Control Group 82								
Pre-intervention		20.38 ± 1.36	37.95 ± 3.80	25.29 ± 2.35	83.62 ± 4.31	43.94 ± 2.18	89.33 ± 3.35	133.27 ± 4.17
Post Intervention		20.71 ± 1.28	38.95 ± 2.84	26.11 ± 2.19	85.77 ± 3.53	44.59 ± 2.37	89.70 ± 3.51	134.28 ± 4.21
Experimental Group 86								
Pre-intervention		20.60 ± 1.37	38.49 ± 3.42	25.51 ± 1.98	84.60 ± 4.13	44.24 ± 2.18	90.16 ± 3.35	134.41 ± 4.36
Post Intervention		24.14 ± 1.72	43.72 ± 1.58	32.84 ± 2.58	100.70 ± 3.80	50.95 ± 2.34	99.12 ± 4.55	141.12 ± 3.59
*t*_1_ value *p*_1_ value *t*_2_ value *p*_2_ value		-1.075	-0.964	-0.653	-1.510	-0.907	-1.613	-1.729
		0.284	0.336	0.514	0.133	0.366	0.109	0.086
		-14.734	-13.352	-18.184	-26.351	-17.547	-15.076	-11.332
		<0.001	<0.001	<0.001	<0.001	<0.001	<0.001	<0.001

Control group: colorectal cancer patients received health education according to the perioperative chemotherapy nursing routine; experimental group: receive health education based on PRECEDE-PROCEED model. *t_1_, P_1_* values: comparison of the 2 groups before intervention, *t_2_, P_2_* values: comparison of the 2 groups after intervention.

### 3.3 Comparison of home health care experience scores between two groups

On the 5th and 10th days after discharge, the experimental group had significantly higher PACT-M2 scores compared with the control group (all *p* < 0.05), indicating better home health care experiences. After the intervention, the effect size for home health care experience at 5 days post-discharge in the intervention group was large (Cohen’s d = 1.688, 95% CI [1.334, 2.039]). The effect size at 10 days post-discharge was even larger (Cohen’s d = 2.354, 95% CI [1.957, 2.746]). Detailed results are presented in **Table 4**.

**Table 4:** Comparison of PACT-M2 scores on the 5th and 10th days after discharge between experimental and control groups.

Category	Number of cases	The 5th day after discharge	The 10th day after discharge
Control group	82	24.73 ± 2.63	26.49 ± 1.86
Experimental group	86	28.63 ± 1.95	31.19 ± 2.12
*t* value		-10.862	-15.248
*p* value		<0.001	<0.001

Missing data for ‘Not Applicable’ items were handled using mean imputation as per the protocol described in Section 1.5.

### 3.4 Comparison of unplanned readmissions between the two groups

Within three weeks after discharge, unplanned readmissions occurred in 2 patients (2.32%) in the experimental group and 4 patients (4.88%) in the control group; this difference was not statistically significant (*p* > 0.05), as presented in **Table 5**.

**Table 5:** Comparison of unplanned readmission rates within three weeks after discharge between experimental and control groups.

Category	Number of cases	Number of unplanned readmissions (cases)	Test statistic	*p* value
Experimental group	86	2(2.32%)	0.794	0.373
Control group	82	4(4.88%)		

## 4 Discussion

### 4.1 Effect of PRECEDE-PROCEED model on discharge readiness in patients receiving first chemotherapy

Readiness for hospital discharge is an important indicator of whether patients can be discharged safely, and efficient discharge readiness contributes to improved quality of life after discharge (8, 27). The results of this study indicated that the experimental group scored significantly higher than the control group in all dimensions of hospital discharge readiness, including personal status, adaptability, and anticipatory support, as well as in the total score (*p* < 0.01). These findings are consistent with results from related studies (28).

The core advantage of the PRECEDE-PROCEED model of health education lies in its systematic, multidimensional intervention approach to patient health behavior. In contrast to the control group, which received only routine health education, the experimental group underwent a phased model beginning with “social diagnosis” to identify key issues faced by patients after the first cycle of chemotherapy, such as insufficient knowledge regarding the management of chemotherapy side effects and lack of confidence in home care.

This was followed by “epidemiological diagnosis” and “behavioral and environmental diagnosis” to accurately identify the factors influencing discharge readiness, including patients’ understanding of home diet, activity, psychological health, recognition of chemotherapy responses, and the capacity of family caregivers to provide support.

Finally, through “educational and organizational diagnosis” and “management and policy diagnosis,” predisposing, enabling, and reinforcing factors were analyzed at different stages of hospitalization to design targeted intervention measures.

These measures included phased knowledge explanations, situational drills to manage adverse reactions to chemotherapy, simulated home environment training, synchronous training for family caregivers, and interactive engagement via an the platform. This stepwise intervention model not only addressed knowledge deficits, but also enhanced the self-care ability of patients, thereby producing comprehensive improvements in readiness for hospital discharge.

### 4.2 Effect of PRECEDE-PROCEED model on the quality of discharge instructions for patients receiving their first cycle of chemotherapy

The quality of discharge instructions refers to the accuracy, relevance, and clarity of medical information provided to patients and their families through face-to-face communication, lectures, multimedia, and other educational methods. High-quality discharge instructions effectively promote the compliance of patients with post-hospital rehabilitation care, encourage regular follow-up, and strengthen self-rehabilitation ability (29). The results of this study indicated that the experimental group had significantly higher scores in all dimensions of discharge instruction quality including actual content, guidance skills, and guidance effects as well as in the total score compared with the control group (*p* < 0.01). These results exceeded those reported in similar studies (30).

Routine health education generally follows a “one-way instillation” approach, in which nurses deliver discharge precautions in a centralized manner (31). This approach often results in complex, non-specific content that patients may forget due to information overload (32). Discharge preparation based on the PRECEDE-PROCEED model overcomes these limitations.

Regarding guidance, stratified instruction was provided according to individual patient differences. For example, professional terminology was simplified for older adults, illustrated manuals and video materials were integrated, and detailed information on chemotherapy drug metabolism was offered to patients with higher education levels. This ensured that the obtained content corresponded more closely to patient needs.

Regarding guidance skills, an interactive “explanation–demonstration–feedback” model was used. Nurses demonstrated port maintenance procedures, patients or family members practiced them, errors were corrected in real time, and comprehension was reinforced through questioning and case analysis. This patient-tailored and interactive approach compensated for the generalization of content and limited scope of skills observed in routine instruction, thereby enhancing the overall quality of discharge guidance.

### 4.3 Effect of PRECEDE-PROCEED model on home health care experience in patients receiving their first cycle of chemotherapy

Comprehensive rehabilitation knowledge and skills can allow patients to address potential health problems after discharge more effectively, thereby enhancing their home health care experience (25). In this study, home health care experience scores were significantly higher in the experimental group on the 5th and 10th days after discharge compared with the control group (*p* < 0.05), indicating that discharge preparation based on the PRECEDE-PROCEED model provided continuous improvement in the post-discharge care experiences of patients.

During the early stage after discharge, patients receiving their first cycle of chemotherapy for colorectal cancer often experience anxiety due to the lack of on-site guidance from medical staff. Furthermore, adverse effects of chemotherapy including nausea and vomiting, fatigue, and diarrhea tend to emerge gradually after discharge, affecting home care experiences.

The PRECEDE-PROCEED model-based discharge preparation approach addressed these challenges through two key strategies. Previous work shows that predisposing-enabling-reinforcing structures improve self-efficacy in first-cycle chemotherapy patients (33). First, during the educational diagnosis stage before discharge, training content was tailored to address high-frequency needs, such as coping with adverse reactions of chemotherapy at home and emergency management. This enabled patients to master specific coping strategies, such as dietary adjustments for constipation or diarrhea, thereby reducing anxiety associated with the unknown. Second, a short-term follow-up mechanism was established, involving telephone follow-up on the 5th and 10th days after discharge and providing responses within two hours to queries received via the platform. This ensured timely answers to patient questions and continuous support. The smartphone app follow-up achieved 75% sensitivity and 83% specificity for detecting postoperative complications within 15 days, with 83.7% patient adherence and an NPS satisfaction score of 94 (34).

Although 3-week unplanned readmissions did not differ statistically (p = 0.373), *post-hoc* power analysis revealed that the study had only 23.4% power to detect a 2.6% absolute difference in readmission rates with the current sample size (two-proportion test, α = 0.05). This suggests the nonsignificant finding likely reflects insufficient statistical power rather than absence of a true effect. The observed 2.6-percentage-point absolute reduction (4.88% vs 2.32%, number needed to treat [NNT] ≈ 38) suggests potential clinical relevance that warrants investigation in adequately powered trials. Longer follow-up periods are needed to determine whether improved discharge readiness translates into sustained reductions in readmissions.

Additionally, the specialist nurses regularly disseminated concise home care knowledge, identified adverse reactions, and shared practical self-care strategies via the platform three times per week. Patients were also reminded via SMS through the platform to record their self-management measures, reinforcing multidimensional self-care knowledge encompassing disease understanding, medication use, diet, psychological health, and rehabilitation exercises. This combination of thorough predischarge preparation and timely post discharge follow-up strengthened patient confidence in home care and improved the overall home health care experience.

### 4.4 Limitations and future directions

Several limitations should be acknowledged. First, as a quasi-experimental study, the use of admission time grouping rather than randomization introduces potential temporal bias, including the possibility of unmeasured confounders varying between January-April and May-August 2025. The short observation period (3 weeks) did not allow assessment of longer-term prognostic indicators such as outcomes at 1 month or 3 months post-discharge. Although we monitored key contextual factors and confirmed stability in chemotherapy protocols, staffing, and hospital policies during the study period, we cannot entirely exclude subtle temporal variations (e.g., patient seasonal characteristics, evolving clinical practices) that may have influenced outcomes. A sensitivity analysis comparing baseline characteristics between early and late enrollees within each group showed no significant differences (p > 0.05 for all variables), supporting the temporal stability of our sample. Future studies should employ cluster randomization or stepped-wedge designs to mitigate this limitation.

Additionally, stratified analysis was not conducted for potential confounding factors, including family caregiver participation and patients’ education levels, which may influence the effects of the intervention. This limits the ability to clarify differential responses to the regimen among distinct patient populations.

Third, the single-center design in a tertiary hospital in Jiangsu Province limits generalizability to other settings, particularly community hospitals, rural areas, or countries with different healthcare infrastructures. Also, the intervention in this study relied on the platform, which may limit applicability to populations with lower digital literacy or limited smartphone access. The homogeneity of our sample (predominantly married, junior high school educated, with health insurance) may not reflect the diversity of the broader colorectal cancer population. Caution should be exercised when applying these findings to older adults, underserved populations, or settings with limited nursing resources. Future implementations should develop alternative delivery modes (e.g., telephone-based follow-up, paper-based educational materials) to enhance inclusivity and evaluate effectiveness across diverse digital access levels.

The fourth limitation is the lack of blinding among outcome assessors represents a threat to internal validity. While we standardized assessment procedures, the involvement of intervention-delivering nurses in outcome measurement may have led to measurement bias, particularly for subjective outcomes such as discharge readiness and home care experience. Future studies should employ independent research assistants, blinded to group allocation, for all outcome assessments.

The fifth limitation is the lack of full independence between intervention providers and outcome assessors. Future multicenter studies should aim to incorporate independent outcome assessment to further reduce the risk of bias.

Future research should conduct a multicenter pragmatic randomized controlled trial employing rigorous randomization and blinding procedures, with an expanded sample size and an extended follow-up period of at least 6 months. Such a design would allow not only evaluation of the sustainability of the intervention effects but also assessment of its impact on adherence to subsequent chemotherapy cycles, quality of life, and long-term clinical outcomes. Intervention providers and outcome assessors should be fully independent to minimize potential bias. Guided by the PRECEDE–PROCEED model, future studies should also develop and apply validated instruments specifically designed to quantify changes in predisposing, enabling, and reinforcing factors, thereby clarifying the mediating effects of each model component within the intervention pathway. In addition, a formal economic evaluation (e.g., cost–effectiveness analysis) should be undertaken to provide empirical evidence regarding the proposed “low-cost, high-benefit” advantages of the program. Such comprehensive evaluation would generate more robust evidence to inform and optimize nursing management strategies for discharge preparation services among patients undergoing first-cycle chemotherapy for cancer.

## 5 Conclusion

This study did not detect a significant between-group difference in unplanned readmission within 3 weeks after discharge. This may be attributable to the multifactorial nature of readmission, which can be influenced by underlying comorbidities, chemotherapy-related toxicity, family caregiving capacity, and other complex factors. In addition, the relatively short follow-up period and limited sample size may have reduced the ability to detect differences in this outcome. Nevertheless, this study successfully demonstrated the feasibility of implementing a PRECEDE–PROCEED–based health education discharge preparation program in patients with colorectal cancer undergoing first-cycle chemotherapy. The findings indicate a strong positive association between the intervention and improvements in readiness for hospital discharge, quality of discharge teaching, and home health care experience. These results suggest that the intervention effectively enhanced key process-related outcomes. Furthermore, this study systematically delineated the structure and implementation pathway of the PRECEDE–PROCEED–based health education program for patients receiving first-cycle chemotherapy for colorectal cancer, thereby providing a practical and reproducible framework for delivering standardized yet individualized discharge education in clinical settings. Overall, this model represents a promising innovation in clinical health education practice and warrants evaluation of its practical value in wider implementation through future randomized studies.

## Data Availability

The original contributions presented in the study are included in the article/supplementary material. Further inquiries can be directed to the corresponding authors.
